# Markers for Characterization of Bone Marrow Multipotential Stromal Cells

**DOI:** 10.1155/2012/975871

**Published:** 2012-05-14

**Authors:** Sally A. Boxall, Elena Jones

**Affiliations:** Academic Unit of the Musculoskeletal Diseases, St. James's University Hospital, Leeds Institute of Molecular Medicine, University of Leeds, Leeds LS9 7TF, UK

## Abstract

Given the observed efficacy of culture-expanded multipotential stromal cells, also termed mesenchymal stem cells (MSCs), in the treatment of graft-versus host and cardiac disease, it remains surprising that purity and potency characterization of manufactured cell batches remains rather basic. In this paper, we will initially discuss surface and molecular markers that were proposed to serve as the indicators of the MSC potency, in terms of their proliferative potential or the ability to differentiate into desired lineages. The second part of this paper will be dedicated to a critical discussion of surface markers of uncultured (i.e., native) bone marrow (BM) MSCs. Although no formal consensus has yet been reached on which markers may be best suited for prospective BM MSC isolation, markers that cross-react with MSCs of animal models (such as CD271 and W8-B2/MSCA-1) may have the strongest translational value. Whereas small animal models are needed to discover the *in vivo* function on these markers, large animal models are required for safety and efficacy testing of isolated MSCs, particularly in the field of bone and cartilage tissue engineering.

## 1. Introduction

BM MSCs were discovered in the late 1970s by a group led by a Russian-born scientist Alexander Friedenstein, who showed that BM contains a population of plastic-adherent, highly proliferative cells, that were able to form colony of fibroblasts (hence the name colony-forming unit-fibroblasts, CFU-F) [1, 2]. Following implantation in diffusion chambers, CFU-Fs spontaneously formed bone, cartilage, and fibrous tissue *in vivo* [3]. Whereas Friedenstein termed them “determined osteogenic progenitors” [4], the subsequent findings of their multipotentiality toward other mesenchymal lineages led Arnold Caplan to coin the term “mesenchymal stem cells” [5], in analogy to “hematopoietic stem cells” (HSC), which were the best described adult stem cell type at the time. 

## 2. Potency Markers of Cultured MSCs

The first definitive markers of MSCs were proposed in a pioneering study of Pittenger et al., the group who also developed robust and reproducible *in vitro* assays of MSC multipotentiality towards bone, cartilage, and fat lineages [6]. These BM MSC markers included SH2 and SH3, later shown to correspond to CD105 and CD73 molecules, respectively [7, 8]. Of note, CD stands for “cluster of differentiation”, the standard nomenclature for cell surface molecules. These two markers alongside CD90 are positively expressed on MSCs and remain the primary molecules used to identify MSCs by the International Society of Cell Therapy (ISCT) position statement [9]. The ISCT position statement also advices that MSCs should be negative for the expression of CD11b or CD14, CD19 or CD79a, CD34, CD45, and HLA-DR [9]. This is primarily to allow the exclusion of haematopoetic cells which may contaminate MSC cultures.

CD105, also known as endoglin, is the TGF-beta receptor III, which potentially plays a role in TGF-beta signalling during MSC chondrogenic differentiation [7]. CD73 is an ecto-5′-nucleotidase, which is known to be involved in BM stromal interactions [8], MSC migration [10], and, potentially, MSC modulation of adaptive immunity [11]. The exact function of the CD90 (Thy1 antigen) is less well defined. It has been proposed to mediate cell-cell interactions [12, 13], involved in adhesion of monocytes and leukocytes to endothelial cells and fibroblasts [14, 15], and may have a role in the stromal adherence of CD34+ cells [16].

Cultured MSCs are uniformly and strongly positive for CD105, CD90, and CD73, regardless of their passage or time in culture [6, 17]. However, CD105 and CD73 are also expressed on skin fibroblasts [18, 19], cells with a much lower ability to proliferate and differentiate, compared to BM MSCs [6, 19]. Furthermore, another plastic-adherent cell type that is able to propagate *in vitro*—umbilical vein endothelial cells—is also CD105 and CD73 positive [20, 21]. This implies that sole demonstration of CD105 and CD73 expression without CD90 on adherent cultured cells is insufficient to prove their MSC identity. Another disadvantage of CD73 and CD105 is a limited cross-reactivity of anti-human antibodies with animal cells (Table 1), an issue that will be discussed later in the paper.

A complication regarding the long-term cultivation of MSCs was raised when Prockop's group showed a reduction in their colony-forming efficiency with increasing passage [22]. Earlier passage MSCs were documented to have better colony-forming efficiency compared to later passages [22]. This phenomenon was shown to be linked with telomere erosion [23] and later described as “*in vitro* MSC ageing” [24]. These ideas were further extended by Wagner et al. who showed that alterations in phenotype, differentiation potential, gene expression, and miRNA patterns “are not restricted to later passages, but are continuously acquired with increasing passage” from the first passage onwards [25]. The fact that CD105, CD73, and CD90 are expressed at similar levels in early-passage (potent) and late-passage (aged, presenescent) MSCs indicates that their value maybe limited only to basic MSC characterization. The limitation of these markers is further demonstrated by the fact that although CD73 and CD105 are expressed on clonally derived MSCs [6], only 1/3 of these clones are truly multipotential [6]. This suggests that CD73 and CD105 expression may not be directly linked with MSC differentiation capacity.

Stro-1 was another molecule described to be highly specific for BM CFU-F [26]. However, the Stro-1 antigen remains unclustered, limiting its widespread use in human and animal experimentation. Interestingly, Stro-1 expression is downregulated during prolonged culture [26]. The function of Stro-1 on MSCs remains largely unknown; in one study, Stro-1^+^-expanded MSCs were reported to have a better homing capacity, compared to expanded Stro-1^−^ MSCs, suggesting its potential role in MSC migration and attachment to extracellular matrix [27]. In 2003, Gronthos et al. refined their CFU-F isolation strategy, with the addition of CD106 (VCAM-1) as another MSC marker [28]. Sorting for double-positive cells (Stro-1+CD106+) yielded cell fractions highly enriched for CFU-F [28]. Similar to Stro-1, CD106 expression appears to decline in MSCs at later passages [17, 29, 30]. In contrast to CD105 and CD73, CD106 expression is also strongly downregulated in MSCs after differentiation to adipo-, osteo-, and chondrocytes, suggesting that it may indeed be a marker of the most potent/undifferentiated cells within expanded MSC cultures [31]. Another recently proposed possibility is that similar to Stro-1, CD106 (VCAM-1) expression on cultured MSCs is also related to their homing, migration, and adhesion capabilities [29].

Based on these and similar published findings, one can conclude that there exist two categories of markers for cultured MSCs. One category includes molecules that are stably expressed *in vitro*, with little difference between donors and little correlation with culture's *in vitro* history and ageing status (such as CD73, CD90, and CD105). The other, “second-tier” group of markers contains molecules which show dependency on donor or culture “age” or any other variables such as cell homing/attachment properties or cell seeding density (such as Stro-1 or CD106). Another example is PODXL, a sialomucin in the CD34 family, which marks highly proliferative MSCs in low-density, low-passage cultures and is downregulated in high-density cultures [33]. It is tempting to speculate that second-tier markers may be reflective of the MSC maturity or potency status at the single-cell level; if this stands true, a combination of markers from both groups will be needed for quality-control of MSC batches with characterized levels of potency. A concerted effort from different laboratories is needed to validate previously reported “second-tier” markers in respect to donor age, culture conditions, and seeding densities and to validate correlations and reproducibility between different centers.

A good example of such joint effort can be illustrated in Wagner et al., where candidate gene expression markers were validated in 4 centers across Europe [34]. High variability between centers was found [34] and the measurement of MSC methylation status was proposed to be a better way of monitoring *in vitro* MSC ageing [34, 35]. Alternatively, the lengths of telomeres in cultured MSCs may serve as a “true” indicator of MSC age in culture. Gradual telomere shortening in cultured MSCs was first documented by Banfi et al. [23] and further demonstrated by Baxter et al. [24] and other independent investigators [17, 36, 37]. It is noteworthy, however, that telomere lengths in human populations are heritable, showing a very high degree of donor-to-donor variability [38]. Similar to “second-tier” surface markers described previously, the utility of telomere length analysis as a measure of MSC “ageing” status may be limited to a single MSC batch at different stages of manufacture, rather than for comparison between batches from different donors. Such analysis may be very useful for bulk manufacture of MSCs for allogeneic use, whereby the rate of telomere erosion between passages can be seen as an indicator of their overall proliferative potency.

What about markers indicative of MSC propensity to differentiate? CD106 was proposed to be such marker by Fukiage et al. who showed that CD106+ BM MSCs were less osteogenic and more adipogenic than CD106-MSCs [39]. In this respect it is noteworthy that MSC proliferative and overall differentiation capacities are known to be intricately linked. It is now broadly accepted that aged, presenescent MSCs have a significantly reduced differentiation capacity towards adipogenic and chondrogenic lineages compared to early-passage MSCs (reviewed in Sethe et al. [40]). This implies that measuring the MSC senescence status can in fact be indicative, to some degree, of their multipotentiality. At the clonal level, it has been recently shown that the most proliferative, tripotential clones are rapidly growing, whereas bi- and unipotential clones expand slower [41]. As early as 2000, Muragia et al. demonstrated that the majority of BM CFU-Fs are in fact unipotential towards osteogenesis [42]. Standard MSC cultures are composed of a mixture of uni-, bi-, and tripotential CFU-Fs and their precise ratio and relative rates of growth, in our opinion, determine the levels of multipotentiality of standard MSC cultures. General decline in MSC multipotentiality during extended passaging seems to correlate well with a known decline in CD106+ cells [17, 29, 30] supporting the idea that CD106 may indeed mark the most immature, multipotent (rather than uni- or bipotent) progenitors. 

## 3. BM MSC Markers in Animal Models

Animal models have become crucial for preclinical testing of MSC preparations. MSCs from larger animals (dog, sheep, goat, and horse) are normally used for a preclinical evaluation of bone and joint tissue regeneration from MSCs [43, 44]. Such large animal models carry significant logistical and financial considerations but can in fact be useful in some cases whereby veterinary patients can be recruited (such as race horses) [45]. Pig is emerging as the species of choice for preclinical evaluation of the immunomodulatory effects of MSC in terms of both cardiac repair [46] and prevention of immune rejection after solid organ transplant [47]. Smaller animals like rats are frequently used for testing neurological and brain injury repair [48]. Mice have been used to study the immunomodulatory properties of MSCs in both autoimmune [49, 50] and neurological [51] disease models. Although mouse models provide proof-of-principle and allow testing of MSC function in a variety of diseases including arthritis [52, 53], they often fail, in our opinion, to adequately mirror the human diseases. Naturally occurring diseases in larger domestic animals can be more suitable as disease models for some human genetic and acquired diseases and could help to define the potential and therapeutic efficiency and safety of stem cells therapies [54].

Defining the phenotype of MSCs from different animal species is complicated by a lack of species-specific antibodies (Table 1). Whilst there is a larger selection of species-specific antibodies for the more commonly used small animals such as mouse and rat, species-specific antibodies for larger animals are less common. In the absence of species-specific antibodies for common MSC-selective markers in large animals, the majority of work to date has been performed using anti-human antibodies which do not always cross-react with these species. It should also be noted that there is an increasing amount of data available on MSC phenotype in various species as determined by immunohistochemistry and cytochemical techniques [55–58], but given the semiquantitative nature of this data this paper will focus on the MSC phenotype as determined by flow cytometry.

Many of the antigens which are known not to be expressed on MSCs in humans, such as CD31, CD34, and CD45, are also absent on MSCs from other species (Table 1). Whilst likely to be a true finding, even negative results need to be interpreted with caution as the cross-reactivity of the anti-human antibody clones used have not always been fully evaluated on the species being investigated. Of the publications considered in this paper (Table 1) only a minority give information on whether the anti-human antibodies being used have been validated for cross-reactivity with the target species. Whilst some investigators state that they have not fully validated the antibody cross-reactivity [59], others have screened antibodies of interest for positive expression by flow cytometry on other cell types from the same species [60, 61]. Some investigators have gone further in their evaluation, by performing western blot and immunoprecipitation experiments on both MSCs and control cell types from the same species [62].

Some of the most consistently expressed markers across species are CD29 and CD44, but since these molecules are expressed by multiple cells types in many tissues [63], their lack of specificity may limit their usefulness as a marker for MSCs. CD44 has been recently proposed to be involved in stem cell pluripotency and mark several types of cancer stem cells [64]; its numerous other functions, including roles in cell-matrix interaction, homing, adhesion, matrix assembly, and apoptosis resistance [64], preclude, in our opinion, its widespread use as a robust marker of MSCs.

As mentioned earlier, the current criteria for human MSCs put emphasis on the positive expression of CD73, CD90, and CD105 [9]; however none of these markers are expressed by all species (Table 1). CD90 shows strong expression in the majority of species tested but is absent on MSCs in goats and sheep. Interestingly, the actual tissue distribution of CD90 expression varies with species [65–68] and in humans CD90 expression is more restricted compared to mice [16, 69, 70]. Furthermore, different strains of mice express two alternative CD90 antigens (CD90.1/Thy1.1 or CD90.2/Thy1.2), which only differ by one amino acid [71]. This puts into question the validity of using anti-human CD90 antibodies for other species since this antigen does not appear to be well conserved. In our opinion, the variable levels or complete lack of expression of CD73, CD105 and CD90 in MSCs from some animal species using anti-human antibodies is likely to indicate a lack of antibody cross-reactivity. Species-specific antibodies would be required to confirm the true expression pattern of these molecules.

Whilst expression of the same antigens on MSCs across different species is not essential for defining useful MSC markers, the advantages it would bring to preclinical evaluation in animal models do make this a desirable consideration. A number of known human MSC markers have yet to be tested in all species (Table 1). CD146, for example, shows consistent strong expression in humans, pigs, and sheep but remains to be tested in the largest animal models such as cows and horses. Some markers such as CD271 and W8-B2/MSCA-1 have been used to prospectively isolate MSCs in humans [90], a subject that will be expanded on later. The lack of expression of CD271 on cultured MSCs from any species is perhaps predictable given that CD271 is downregulated on culture of human MSCs [55, 91, 92]. This raises the possibility that the best markers for identification may be different between freshly isolated and culture-expanded cells. Given that expression is observed in most of the species tested, W8-B2/MSCA-1 is an interesting candidate for further investigation. The consistent but low expression (Table 1) could be due to its low-level, homogenous expression on all cultured MSCs or due to a small, but distinct proportion of W8-B2 positive cells within animal MSC cultures; this is something to be considered in future studies addressing W8-B2 expression in MSCs from other animal species such as mouse, rat, and horse.

## 4. Markers for Prospective Isolation of BM MSCs in Humans and Animals

The establishment of robust markers for prospective isolation of MSCs is of utmost importance. Firstly, it is needed to put MSCs on the same footing as HSCs, in which the *in vivo* phenotype is well established [93] allowing the direct study of the function of uncultured HSCs in animal models [94]. Secondly, if the phenotype of plastic-adherent culture-initiating MSCs was known, the contribution of other adherent cells from the marrow (hematopoietic progenitors, monocytic-, and endothelial-lineage cells) to MSC “plasticity” and other characteristics would have been much clearer. Additionally, freshly isolated MSCs that have not been artificially “aged” in culture are likely to have higher multipotential and proliferative capacities compared to their culture-expanded progeny. Finally, MSC cultures established from the selected native MSCs free of contaminating (and potentially inhibitory) plastic adherent cells may have stronger immunosuppressive and lymphohematopoietic engraftment-promoting properties, as shown recently [95]. Stronger immunosuppressive effects may at least in part be mediated by an enhanced support of highly suppressive naive T-regulatory cells [96].

The up-to-date list of candidate markers used to isolate human BM MSCs has been extensively reviewed elsewhere [92, 97, 98]. Here we will discuss several issues that have not been previously highlighted: firstly, the cross-reaction of these candidate markers with other BM cells. As seen in Table 2, almost every previously proposed human BM MSC marker is also expressed on other cell types found in the marrow, be it of hematopoietic or endothelial lineage. This does not pose a significant problem in current MSC manufacture protocols, in which MSCs undergo several rounds of passaging, leading to a gradual loss of these contaminating cells. However if one considers manufacture of MSC-seeded scaffolds in rotating bioreactors [99], adherent contaminating cells may by highly unfavorable, taking up the space and oxygen from growing MSCs. The same considerations apply when freshly purified, uncultured MSCs are used. In one clinical study BM MSCs were concentrated using a commercial concentrator device and it was found that a graft containing >1000 CFU-F/cm^3^ was effective in healing nonunion fractures following percutaneous injection [100]. However it was noted that transplanted MSCs had to compete with other transplanted cells for oxygen and “one way to optimize cell survival is to limit the transplanted cells to those that contribute to the formation of bone (i.e., exclude all others)” [100].

The depletion of undesirable cells and hence an enrichment of human BM MSCs can be achieved by positive selection with markers having the least cross-reactivity with other cell types (Table 2). Notably, CD271 and W8-B2/MSCA-1 have an additional advantage of being highly conserved between species (Table 1) making them usable for fresh MSC isolation in large animals. Specifically, CD271 was found useful for the isolation of BM MSCs in bovine [74] and porcine [81] models. The function of CD271 on MSCs remains incompletely understood. In human jaw periosteum-derived cells, CD271+ and CD271− populations were shown to differ in their mineralizing capacities suggesting that CD271 could “be considered an early surface marker of osteogenic capacity” [121]. In dental pulp stem cell cultures, CD271 was proposed to have a role in inhibiting their differentiation [122]. In another view, CD271 is a general neural crest stem cell marker [123], thus putatively explaining its expression on melanoma cancer stem cells [123] and, potentially, on native follicular epithelial cells [124].

W8-B2/MSCA-1 antigen is identical to tissue nonspecific alkaline phosphatase (TNAP), an enzyme known to be expressed at high levels in human liver, bone, and kidney and in embryonic stem cells [125, 126]. In a knockout mouse model, TNAP was shown to promote bone mineralization by providing free inorganic phosphate and by degrading inorganic pyrophosphate, which inhibits mineralization [127]. The STRO-3 antibody has also been shown to bind to TNAP and be a good marker of uncultured BM MSCs [128]. Notably, we demonstrated the expression of bone/liver alkaline phosphatase on the surface of BM CD271+ cells in 2006 [92].

**Figure 1:**
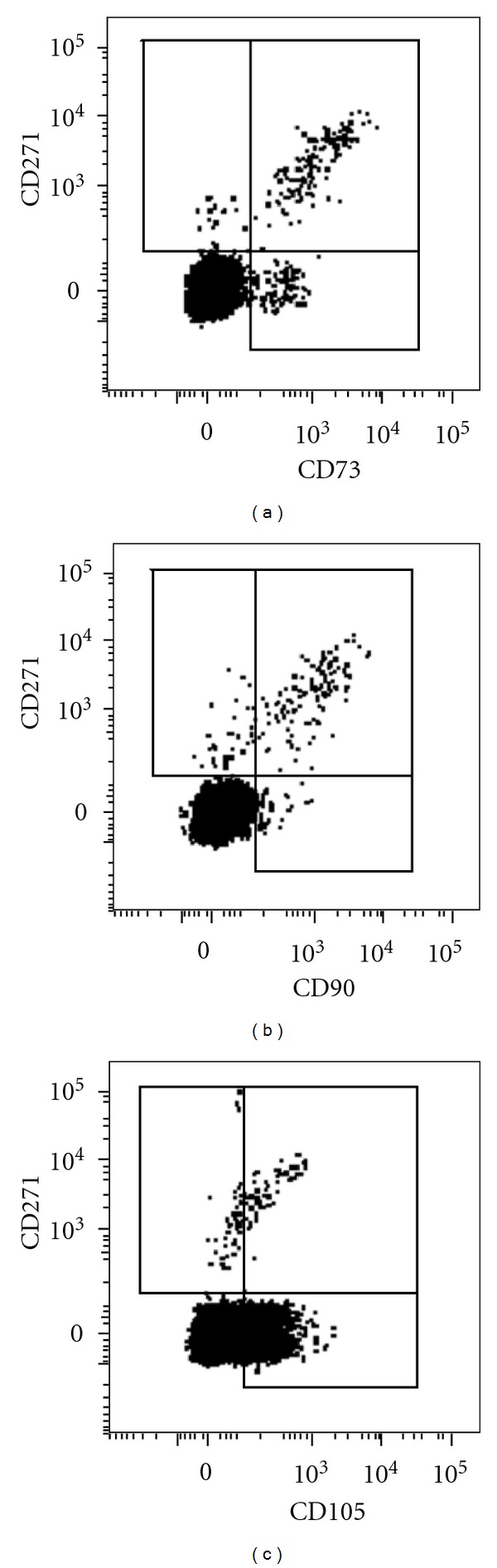
Coexpression of (a) CD271/CD73, (b) CD271/CD90, and (c) CD271/CD105 on CD45-/low cells in human bone marrow aspirates determined by flow cytometry. Mononuclear cells were isolated from bone marrow aspirates and stained with antibodies as previously described [32].

It is generally accepted that the sole positive selection for CD271 may not be sufficient to achieve 100% purity for human MSCs. The removal or “gating out” of hematopoietic lineage cells is commonly required, as CD271 is expressed at low levels on hematopoietic progenitor cells of the erythroid lineage [57, 129]. When gated only on the non-hematopoietic (CD45−/low fraction), the human BM MSC population (CD73+CD105+) can be easily found [56]. In our hands however, CD105 appears to be less discriminative than CD271, CD73, or CD90 (Figure 1) indicating that CD271 and/or CD73 gating is possibly the best way for identifying and sorting human BM MSCs to the highest levels of purity [129, 130]. Several other studies have been performed recently aimed at achieving high-purity BM MSCs using a combination of CD271 and markers other than CD73, CD105, or CD90. For example, CD146 has attracted a lot of interest recently, based on seminal papers by Sacchetti et al. [131] and Crisan et al. [132] linking CD146 expression on MSCs with their pericyte topography and function. More recently however, it was shown that CD146 expression on CD271+ MSCs correlates more with their *in situ* localization [57] and/or the age of donor [133]. Perhaps more promising for the refinement of the MSC purification strategy to 100% purity would be further selection for W8-B2/MSCA-1 expression [90, 98]; these findings are awaiting further confirmation by other independent investigators.

## 5. Molecular Markers of Purified Uncultured MSCs

It would be advantageous if a molecular marker of MSCs, in the manner of oct-4 for embryonic stem cells [134], could be found, helping to identify MSCs in other tissues and organs. To date, this has proven elusive. Instead, the simultaneous expression of transcription factors (TFs) triggering several mesenchymal lineages (including, but not limited to, adipogenic, chondrogenic, and osteogenic) has been reported in native BM MSCs [28, 57] or their expanded progeny [135]. We have recently found strong expression of pericytic and hematopoiesis-supportive genes in CD271+ BM MSCs [136], confirming and extending earlier findings obtained using Stro-1 based MSC selection [137]. We additionally observed prominent Wnt pathway signaling activity in uncultured BM MSCs, which was significantly stronger compared to cultured MSCs or skin fibroblasts [136]. Further advance in qPCR methodology allowing the simultaneous assessment of thousands of candidate genes in rare, sorted MSCs is likely to reveal novel gene(s) with robust, strong expression and high selectivity. These new gene transcripts could be used as molecular markers of marrow MSCs leading to clear demonstration of their in vivo function using knock-out animal models.

## 6. Concluding Remarks

At present, we cannot definitely conclude that MSCs resident in different tissues are the same or even very similar. For example, adipose-derived MSCs express CD34 [138] whereas BM MSCs do not (Table 1). CD271 is expressed in the synovium [139], but the phenotype of synovial MSCs may be much broader [140]. W8-B2/MSCA-1 is expressed by BM MSCs but not placenta-derived MSCs [141]. This suggests that the search for novel markers, intricately linked to the fundamental MSC function, including both surface and molecular markers, should continue. The knowledge of the phenotype and gene expression profile of BM MSCs in their original niche should undoubtedly help to develop new methodologies for expanding these MSCs “in their native state”, via the design of novel biomimetic scaffolds, surfaces, attachment molecules or cytokine cocktails. This is likely to yield MSC-based therapeutic products with significantly improved quality and predictable biological behaviors. Testing of novel purified and expanded MSC-based products in large animal models will allow through pre-clinical evaluation of novel products prior to clinical trials in humans. Additionally, a broader knowledge of native BM MSCs in diseases related to bone physiology and blood cell development, including osteoporosis and leukemias, will lead to a much better understanding of the role of MSCs in the development of these diseases, potentially identifying new targets for therapy.

## Figures and Tables

**Table 1:** Surface antigen expression on cultured MSCs from different species.

Surface antigen	Human	Mouse**	Rat	Rabbit	Primate	Dog	Pig	Goat	Sheep	Cow	Horse
CD13	++ [72]	− [73]*			NC [72]	NC [72]	NC [72]	NC [72]	NC [72]	NC [74]	
	++ [75]	+ [76]*									
	++ [77]										

CD29	++ [72]	++ [78]*	++ [79]*	++ [80]	NC [72]	NC [72]	NC [72]	NC [72]	NC [72]		++ [59]
	++ [81]	++ [73]*	++ [82]*			+ [83]	++ [81]		++ [84]*		++ [62]*
	++ [6]		++ [58]*				++ [46]		++ [85]*		
	++ [75]						++ [47]				
	++ [77]										

CD31	− [72]	− [78]*			NC [72]	NC [72]	NC [72]	NC [72]	NC [72]		
	− [81]	− [86]*					− [81]		− [84]*		
	− [75]						− [46]		− [85]*		

CD34	− [72]	− [78]*		+− [80]	NC [72]	NC [72]	NC [72]	NC [72]	− [61]		−(CND) [59]
	− [81]	+− [73]*			− [87]	− [83]*	− [81]		NC [72]		
	− [6]	− [76]*									
	− [9]	+ [86]*									

CD44	++ [72]	++ [78]*	+ [82]*		NC [72]	NC [72]	NC [72]	NC [72]	++ [61]	++ [74]	− [59]
	++ [81]	++ [73]*				+ [83]*	++ [81]		NC [72]		+ [62]*
	++ [6]						++ [46]		++ [84]		
	++ [77]								++ [85]		

CD49e	++ [81]	++ [73]*	++ [79]*				− (CND) [81]				
	++ [77]										

CD45	− [72]	− [78]*	− [79]*		− [87]	NC [72]	NC [72]	NC [72]	− [61]*	+− [74]*	− (CND) [59]
	− [81]	+− [73]*	+− [82]*			− [83]*	− [81]*		NC [72]		
	− [6]	− [76]*	+− [58]*				− [47]		− [84]		
	− [9]	− [86]*									

CD73	++ [72]	+− [73]*	++[79]*		++ [72]	− [72]	− [72]	− [72]	− [72]	NC [74]	− [59]
	++ [9]		++[88]*								
	++ [75]										
	++ [77]										

CD90	++ [72]	++ [73]*	++[79]*		++ [72]	− [72]	++ [72]	− [72]	− [72]		++ [59]
	++ [81]	+ [76]*	++[82]*			+ [83]	++ [81]				
	++ [6]	− [86]*	++ [58]*				++ [46]				
	++ [9]		++ [88]*				++ [47]				
	++ [77]										

CD105	++ [72]	+ [78]*	++ [79]*		++ [72]	− [72]	− [72]	− [72]	+− [61]	NC [74]	− [59]
	++ [81]	++ [73]*			+ [87]	− [83]	NC [81]		− [72]		
	++ [6]						++ [47]				
	++ [9]										
	++ [77]										

CD146	++ [72]				++ [72]	+ [72]	++ [72]	− [72]	++ [72]		
	++ [81]						+ [81]				
	++ [77]										

CD166	++ [81]	+− [73]*					− [81]		++ [85]	++ [74]	
	++ [75]										
	++ [77]										

CD271	+− [72]				+ [72]	+− [72]	+− [72]	+− [72]	+− [72]		
	+− [81]						+− [81]				

c-Kit (CD117)	− [81]	++ [78]*					− [81]				
		+− [73]*									
		− [76]*									
		− [86]*									

Sca-1	− [72]	++ [78]*			− [72]	− [72]	− [72]	− [72]	− [72]		
	− [81]	++ [73]*					− [81]				
		++ [76]*									
		++ [86]*									

SSEA4	++ [72]	++ [89]*			++ [72]	− [72]	− [72]	+ [72]	− [72]		
	++ [81]						NC [81]				
	++ [89]										
	++ [77]										

Stro-1	++ [81]		++[79]*				+− [81]		− [85]		
	++ [26]										

W8-B2/ MSCA-1	+ [72]				+ [72]	+ [72]	+ [72]	++ [72]	+− [72]		
	+ [81]						+ [81]				

*Species-specific antibody (all others are antihuman antibodies). NC: no cross-reactivity; CND: cross-reactivity not determined. Symbols indicate marker expression levels: −: no expression; +−: <5% expression; +: 5−50% expression, ++: 50−100% expression. **Markers specific for MSCs and MPCs are included due to confusion in terminology.

**Table 2:** Expression of common MSC markers on other cell types found in human bone marrow.

	CD13	CD29	CD44	CD73	CD90	CD105	CD106	CD146	CD200	CD271	STRO-1	SSEA-4
Mesenchymal Stromal Cells	+ [91]	+ [101]	+ [102]	+ [6]	+ [9]	+ [6]	+ [90]	+ [103]	+ [77]	+ [91]	+ [26]	+[89]

Haematopoietic Stem Cell			+ [104]		+ [16]							

Lymphoid lineage		+ [105]	+ [106]	+ [107]					+ [108]			

Myeloid lineage	+ [109]	+ [105]	+ [110]			+ [111]						

Megakaryocytic lineage		+ [112]										+ [113]

Erythroid lineage			+ [110]							+ [57]	+ [26]	+ [114]

Endothelial lineage cells	+ [115]	+ [116]	+ [110]	+ [21]		+ [117]	+ [118]	+ [103]	+ [108]		+ [119]	+ [120]
